# Performance of TGSE BLADE DWI compared with RESOLVE DWI in the diagnosis of cholesteatoma

**DOI:** 10.1186/s12880-020-00438-7

**Published:** 2020-04-19

**Authors:** Yaru Sheng, Rujian Hong, Yan Sha, Zhongshuai Zhang, Kun Zhou, Caixia Fu

**Affiliations:** 1 Department of Radiology, Eye & ENT Hospital of Fudan University, 83 Fenyang Road, Shanghai, 200031 China; 2 Scientific Marketing, Siemens Healthcare, Shanghai, China; 3 Department of Digitalization, Siemens Shenzhen Magnetic Resonance, Ltd., Shenzhen, China

**Keywords:** Cholesteatoma, Middle ear, Magnetic resonance imaging, TGSE BALDE DWI, RESOLVE DWI

## Abstract

**Background:**

Based on its high resolution in soft tissue, MRI, especially diffusion-weighted imaging (DWI), is increasingly important in the evaluation of cholesteatoma. The purpose of this study was to evaluate the role of the 2D turbo gradient- and spin-echo (TGSE) diffusion-weighted (DW) pulse sequence with the BLADE trajectory technique in the diagnosis of cholesteatoma at 3 T and to qualitatively and quantitatively compare image quality between the TGSE BLADE and RESOLVE methods.

**Method:**

A total of 42 patients (23 males, 19 females; age range, 7–65 years; mean, 40.1 years) with surgically confirmed cholesteatoma in the middle ear were enrolled in this study. All patients underwent DWI (both a prototype TGSE BLADE DWI sequence and the RESOLVE DWI sequence) using a 3-T scanner with a 64-channel brain coil.

Qualitative imaging parameters (imaging sharpness, geometric distortion, ghosting artifacts, and overall imaging quality) and quantitative imaging parameters (apparent diffusion coefficient [ADC], signal-to-noise ratio [SNR], contrast, and contrast-to-noise ratio [CNR]) were assessed for the two diffusion acquisition techniques by two independent radiologists.

**Result:**

A comparison of qualitative scores indicated that TGSE BLADE DWI produced less geometric distortion, fewer ghosting artifacts (*P* < 0.001) and higher image quality (*P* < 0.001) than were observed for RESOLVE DWI. A comparison of the evaluated quantitative image parameters between TGSE and RESOLVE showed that TGSE BLADE DWI produced a significantly lower SNR (*P* < 0.001) and higher parameter values (both contrast and CNR (*P* < 0.001)) than were found for RESOLVE DWI.

The ADC (*P* < 0.001) was significantly lower for TGSE BLADE DWI (0.763 × 10^− 3^ mm^2^/s) than RESOLVE DWI (0.928 × 10^− 3^ mm^2^/s).

**Conclusion:**

Compared with RESOLVE DWI, TGSE BLADE DWI significantly improved the image quality of cholesteatoma by reducing magnetic sensitive artifacts, distortion, and blurring. TGSE BLADE DWI is more valuable than RESOLVE DWI for the diagnosis of small-sized (2 mm) cholesteatoma lesions. However, TGSE BLADE DWI also has some disadvantages: the whole image intensity is slightly low, so that the anatomical details of the air-bone interface are not shown well, and this shortcoming should be improved in the future.

## Background

Cholesteatoma is a benign, gradually expanding and destructive epithelial lesion of the temporal bone that results in the erosion of adjacent bony structures and can lead to various complications [1]. In addition to its clinical features and otoscopic findings, the early diagnosis of cholesteatoma based on an imaging examinations, such as high-resolution computed tomography (CT) and magnetic resonance imaging (MRI), is important. From a surgical perspective, high-resolution CT remains the primary imaging technique for the diagnosis and characterization of cholesteatoma in the middle ear due to its high spatial resolution and good visualization of bone structures [2, 3]. However, in terms of its disadvantages, it is difficult to distinguish cholesteatoma from granulation tissue, fibrous tissue, or fluid on high-resolution CT [4]. Based on the high resolution of soft tissue, MRI has gained increasing importance in the evaluation of cholesteatoma. Many studies have shown that MR diffusion-weighted imaging (DWI) has high sensitivity and specificity for identifying the presence of cholesteatoma due to its high keratin content [5–7]. However, conventional DWI uses an echo-planar imaging (EPI) trajectory to collect k-space data, and the obtained images (single-shot echo-planar DWI, SS EPI) may suffer from severe susceptibility artifacts at air-bone interfaces. Additionally, its image resolution is limited. Thus, it is difficult to use DWI in cases in which the lesion is closer than 5 mm [8, 9] from the distortion area. Compared with the EP DWI sequence, the non-echo-planar diffusion weighted imaging (non-EPI) DW imaging sequence produces thinner slices and has a higher imaging matrix, and it tends to produce fewer magnetic susceptibility artifacts but requires longer imaging times (multi-shot non-echo-planar DWI sequences require approximately 8 min), and non-EPI has higher sensitivity for detecting cholesteatoma and a lower misdiagnosis rate [7, 10–12].

Readout-segmented echo-planar imaging (RESOLVE) has been proposed to reduce image distortion. This method could significantly improve head and neck DWI by reducing echo spacing. Although RESOLVE DWI has a significantly improved image signal-to-noise ratio (SNR) and reduced image distortion, the partial volume effect and T2* blurring effect are not completely eliminated, and it is difficult to detect small lesions (< 2.5 mm) [13–15].

Periodically rotated overlapping parallel lines with enhanced reconstruction (PROPELLER) DWI is a nonecho planar fast spin-echo-based DWI sequence in which the central k-space is acquired in a rotating manner. The PROPELLER sequence is free of geometric distortion and susceptibility artifacts, but the scan time is long and imposes a high specific absorption rate (SAR), especially at high fields [16–19].

The BLADE DWI technique has previously been reported to eliminate susceptibility artifacts by applying the ‘blades’ acquisition scheme in k-space [20]. This non-EPI technique was further optimized by using a TGSE method to increase the SNR efficiency and achieve the detection of small (< 2.5 mm) cholesteatomas while increasing the resolution to decrease susceptibility artifacts, thereby allowing differentiation from granulation tissue [20]. Houchun H. et al. [21] concluded that TGSE BLADE DWI exhibited less geometric distortion in the brain and reduced magnetic artifacts near the air-tissue interface than were achieved by conventional SE-EPI. However, the use of TGSE BLADE DWI in studying ear lesions has not yet been reported. L.M.J. Lips et al. [22] found that applying non-EPI DWI for the detection of residual or recurrent cholesteatoma achieved better results at 3 T than at 1.5 T. Hence, the purpose of this study was to evaluate the role of the TGSE BLADE DWI technique in the diagnosis of cholesteatoma at 3 T and qualitatively and quantitatively compare image quality between TGSE BLADE and RESOLVE protocols with the same scanning time (3 m46s).

## Methods

### Patients

In this study, a total of 42 patients (23 males, 19 females; age range, 7–65 years; mean, 40.1 years) with surgically confirmed cholesteatoma were enrolled, and patients with congenital cholesteatoma have been excluded according to the clinical diagnosis from October 2018 to April 2019. Of the 42 patients, 37 had cholesteatoma in the middle and 5 had cholesteatoma in the external auditory canal. Clinicopathological results were the gold standard for all patients.

### Ethics approval and consent to participate

The study was approved by the Review Committee of Eye & ENT Hospital of Fudan University, and written informed consent was obtained from all patients.

### Imaging technique

All patients underwent DWI (both a prototype TGSE BLADE DWI sequence and a commercially available RESOLVE DWI sequence) using a 3 T scanner (MAGNETOM Prisma, Siemens Healthcare, Erlangen, Germany) with a 64-channel brain coil. However, especially for small attic lesions, a golden standard has not been reached at present and axial plane is considered acceptable.

The parameters for TGSE BLADE DWI were as follows: TR/TE = 4000/62 ms; slice thickness/gap = 2/0.2 mm; slices = 21; bandwidth = 520 Hz/Px; field of view (FOV) = 280 × 280 mm^2^; matrix = 192 × 192; voxel size = 1.5 × 1.5 × 2.0 mm^3^; number of excitations (NEX) = 1; diffusion mode = 4 scan trace; b = 0, 1000 s/mm^2^; turbo factor = 13; EPI factor = 3; and data acquisition time = 3 min 46 s. For RESOLVE DWI, the imaging parameters were as follows: TR/TE = 5020/53 ms; slice thickness/gap = 2/0.2 mm; slices = 21; bandwidth = 766 Hz/Px; FOV = 230 × 230 mm; matrix = 192 × 192; voxel size = 1.2 × 1.2 × 2.0 mm; diffusion mode = 4 scan trace; b = 0, 1000 s/mm^2^; and data acquisition time = 3 min 46 s.

### Image assessment

#### Qualitative analysis of image quality

All images obtained in the 42 included patients by TGSE or RESOLVE were evaluated by two radiologists with 10 years of experience in ear MRI evaluation.

Each observer randomly and blindly evaluated the images without knowing the type of DWI sequence and compared the two different DWI sequences using the side-by-side display method. A final decision was made based on mutual consultation when there was a divergence in the assessment results.

**Fig. 1:**
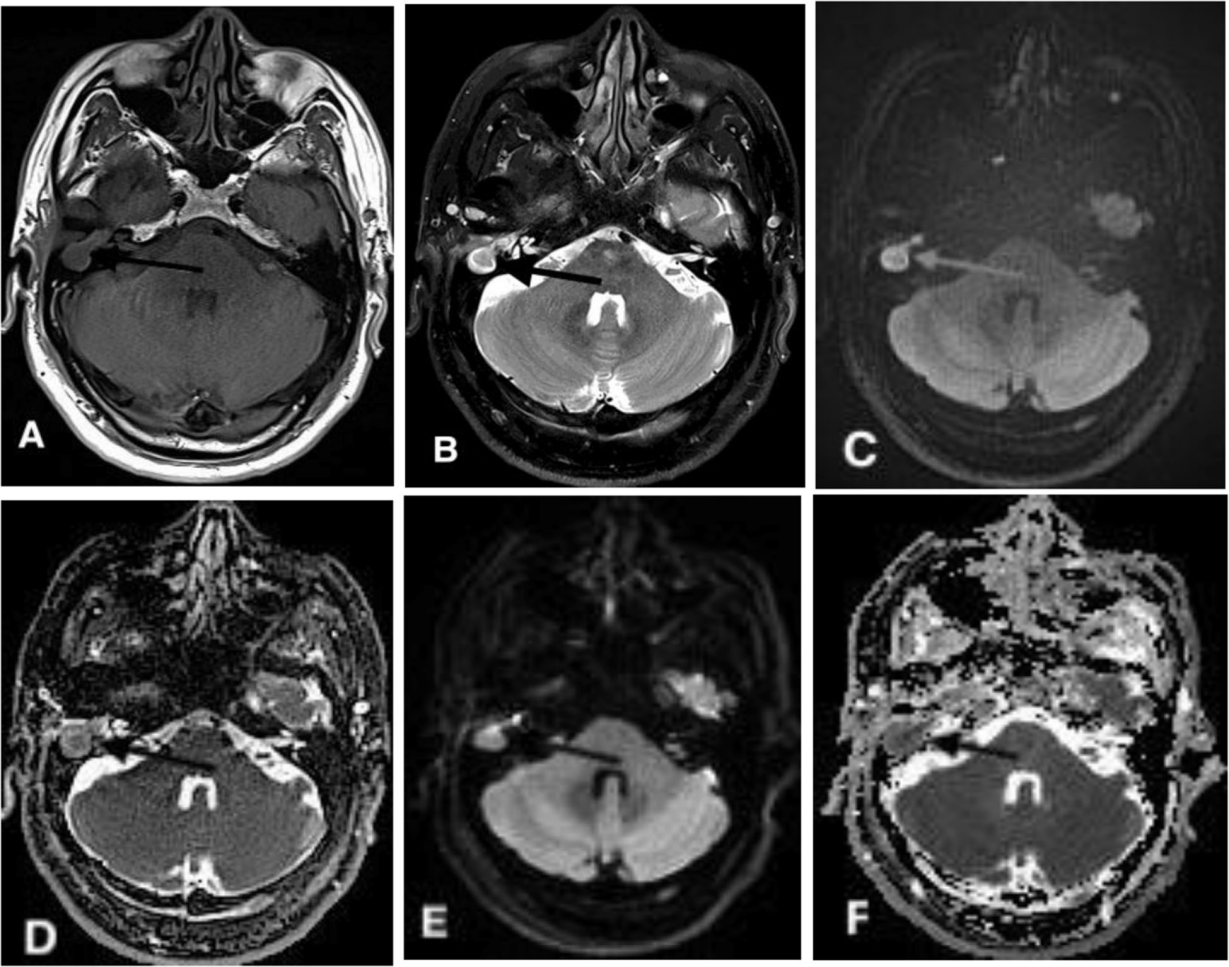
A 51-year-old male with primary cholesteatoma in the right middle ear confirmed by right mastoidectomy. Axial T1WI (**a**) and T2WI (**b**) showed the anatomical location of the cholesteatoma (arrow) in the right middle ear. **c**, **e**: TGSE BLADE (b1000) and RESOLVE (b1000) DWI showed a restricted diffusion lesion (high signal) in the right mastoid bone. **d**, **f**: The ADC on TGSE BLADE and RESOLVE DWI was 0.689 × 10^− 3^ mm^2^/s and 0.791 × 10^− 3^ mm^2^/s, respectively. However, the cholesteatoma lesion (arrow) was not as clear on RESOLVE DWI as on TGSE DWI. Moreover, the structures of the nasal cavity were obviously distorted on the RESOLVE b1000 (**e**) and ADC (**f**) maps, whereas almost no distortion was observed on the TGSE b1000 and ADC maps. Images (**c** and **e**) show the geometric deformation values of the entire image for images with a score of 4 (no distortion) and 2 (moderate distortion)

Qualitative evaluation of images obtained by TGSE and RESOLVE was performed according to four criteria: image sharpness, geometric distortion, ghosting artifacts, and overall image quality. Image sharpness was assessed on a scale from 1 to 3. Both geometric distortion and ghosting artifacts were evaluated on a scale from 1 to 4, and the evaluation of geometric distortion included two parts: the whole image and the lesion in the ear. Overall image quality was also graded on a scale of 1 to 5. The detailed qualitative evaluation criteria are shown in Table 1. In Fig. 1, images C and E show geometric deformations with a score of 4 (no distortion) and 2 (moderate distortion), respectively.

**Table 1:** Qualitative criteria for comparing image quality of TGSE and RESOLVE (b1000) sequences

Image sharpness (anatomical structures: nasal sinus, ear, brain)	
1. Completely unclear 2. Generally visible 3. Clearly visible	
Geometric distortion ① Cholesteatoma (size and border) ② Whole image 1. Severe distortion 2. Moderate distortion 3. Mild distortion 4. No distortion	
Ghosting artifacts (interface of cholesteatoma and the temporal lobe) 1. Severe artifact, unable to distinguish lesions and normal tissues 2. Moderate artifact, part of the lesion can be distinguished from normal tissues 3. Mild artifact, no impact on lesion diagnosis 4. No artifact	
Overall image quality 1. Nondiagnostic 2. Barely diagnosis 3. Diagnostic 4. Good 5. Excellent

#### Quantitative analysis of image quality

The SNR, contrast and contrast-to-noise ratio (CNR) were the main evaluation criteria for the quantitative analysis of images obtained using the two sequence. The apparent diffusion coefficients (ADCs) of the two sequences were compared simultaneously. On the b1000 TGSE and RESOLVE images, the SNR of the lesions in the region of interest (ROI) was defined as the ratio of the mean signal intensity of the lesion (S_ROI_) to the standard deviation of the background noise (σ_BG_) (SNR = S_ROI_/σ_BG_) [23]. The SNR of the brainstem was calculated by the same method as follows: SNR = S_B_/σ_BG._ Contrast was defined as the ratio of the signal intensity of the lesion (S_ROI_) to the signal intensity of the brainstem (S_B_) on the b1000 map (contrast = S_ROI_/S_B_).

The CNR was defined as the difference between the S_ROI_ and S_B_ divided by the standard deviation in the lesion ROI (σ_ROI_) and the brainstem ROI (σ_B_) [13–15, 24], as follows:

$$ CNR=\frac{S_{ROI}-{S}_B}{\sqrt{{\sigma_{ROI}}^2+{\sigma_B}^2}} $$

The ROI of the lesion on the b1000 and ADC maps was manually drawn as 1 mm^2^ at the level of the maximum diameter of the lesion, and the corresponding signal intensity and standard deviation were automatically generated on the MRI workstation. The ROI of the brainstem was defined by selecting 10 mm^2^ of the brainstem, and the signal intensity and standard deviation of each ROI were automatically generated. A circular ROI of 10 mm^2^ was set in the background on the b1000 map for all patients, and the standard deviation of the ROI was automatically generated. In selecting the ROI, areas affected by susceptible artifacts or volume effects were avoided.

The parameters were measured independently and randomly by the two raters at an interval of 2 weeks. The mean value of the two measurements was selected as the final data for further analysis. For brain tissue evaluated on DWI sequences, the diagnostic criterion for cholesteatoma was a very high signal intensity that corresponded to limited diffusion on the ADC maps [8, 25]. The sizes of all lesions were determined on T2-weighted imaging (T2WI) based on the size and location of the lesions observed on the TGSE and RESOLVE sequence images and the premise of avoiding artifacts at air-bone interfaces as much as possible.

### Statistical analysis

All statistical analyzes and plots were performed and created using the SPSS 24.0 software package (Chicago, IL, USA), and *P* < 0.05 was considered statistically significant. The normality of all measurements obtained using the TGSE and RESOLVE sequences was tested using the Shapiro-Wilk test. Significant differences in qualitative parameters between TGSE and RESOLVE DWI were determined using the Wilcoxon rank-sum test, and significant differences in quantitative parameters were determined using the paired t-test. The interreader correlation of the ADC as a quantitative index was evaluated using the intraclass correlation coefficient (ICC). The range of the ICC coefficient was set from 0 to 1.00, and the ICC was defined as follows: < 0.40, poor; 0.41–0.60, moderate; 0.61–0.80, good; > 0.81, excellent [26, 27]. The mean ADCs of the lesions and brainstem measured by the two observers were further calculated, and the differences between them were assessed by paired t-test.

## Results

The lesions were clearly displayed on the TGSE and RESOLVE sequence images obtained in 40 patients among the 42 cases. In only two cases, the RESOLVE sequence images produced more magnetic susceptibility artifacts because the lesion was too small (1.9 mm), and it was difficult to distinguish the lesions from the artifacts, while the TGSE sequence images showed the lesions clearly (Fig. 4).

### Qualitative analysis of image quality

**Fig. 2:**
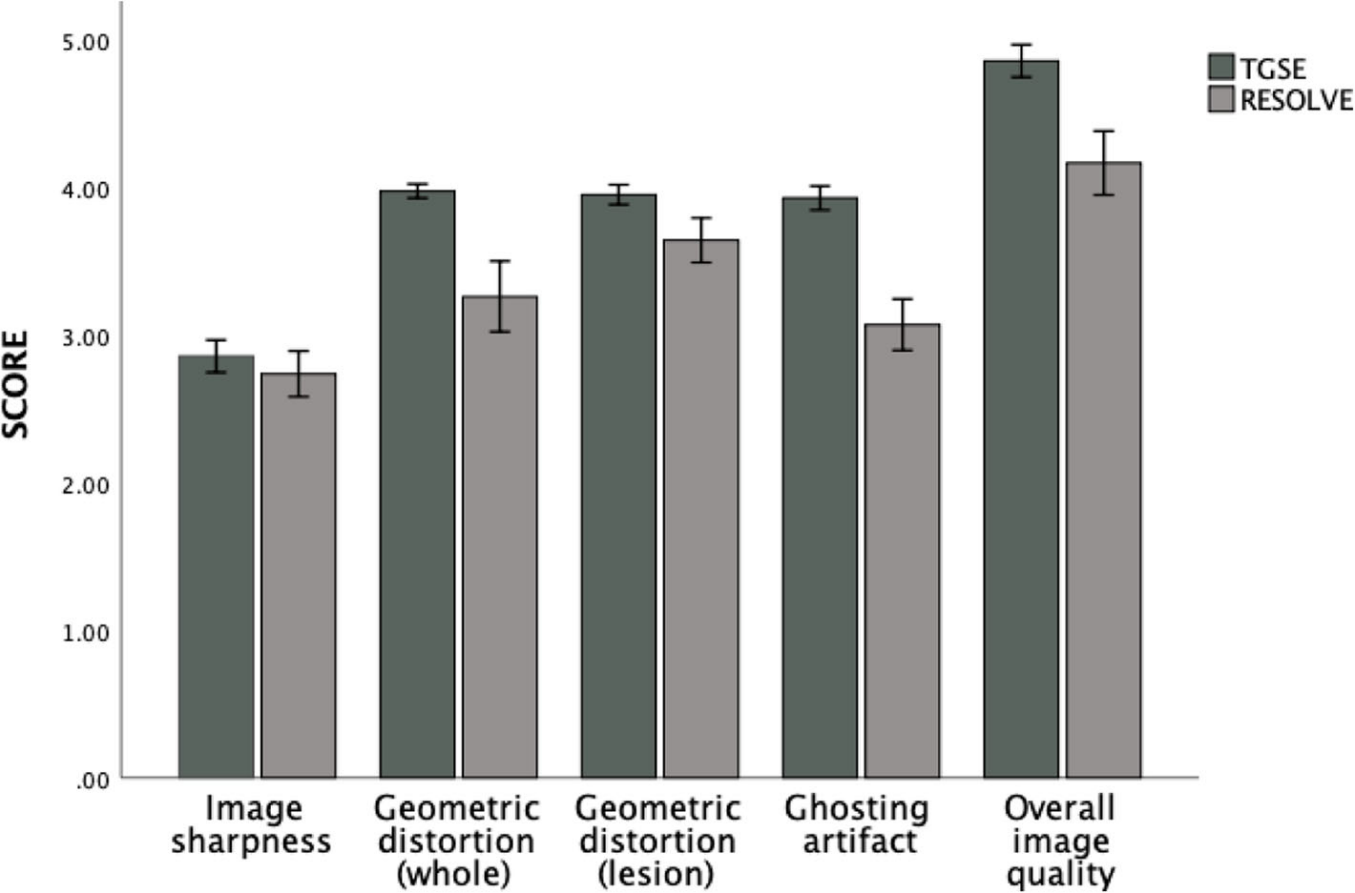
Bar chart showing a comparison of the qualitative imaging parameters between TGSE BLADE and RESOLVE DWI

Comparison of the qualitative scores indicated that TGSE BLADE DWI produced less geometric distortion and ghosting artifacts (*P* < 0.001) and had higher image quality (P < 0.001) than were found for RESOLVE DWI. The average TGSE and RESOLVE scores were as follows (Table 2): geometric distortion (whole), 3.97 ± 0.15 and 3.26 ± 0.26, respectively (*P* < 0.001); geometric distortion (lesion), 3.95 ± 0.21 and 3.64 ± 0.48, respectively (*P* < 0.001); ghosting artifacts, 3.92 ± 0.26 and 3.07 ± 0.55, respectively (*P* < 0.001); and overall image quality, 4.85 ± 0.35 and 4.16 ± .69, respectively. Both TGSE BLADE DWI and RESOLVE DWI had nearly perfect image sharpness (*P* = 0.23; 2.85 ± 0.35 versus 2.73 ± 0.49). Table 1 shows a comparison of the qualitative parameter scores for TGSE BLADE DWI and RESOLVE DWI, and Fig. 2 shows the distributions of the qualitative scores obtained using TGSE BLADE DWI and RESOLVE DWI. As shown in Fig. 1, axial TGSE DWI precisely defined the cholesteatoma lesion in the right middle ear without geometric distortion or ghosting artifacts, whereas RESOLVE DWI showed significant artifacts at the air-bone interface (between the mastoid, i.e., the location of the cholesteatoma lesion, and the nasal sinus).

**Table 2:** Comparison of results of qualitative parameter evaluation between TGSE and RESOLVE images (42 patients)

Parameters	TGSE	RESOLVE	*P* value
Qualitative parameters			
1.Imaging sharpness	2.85 ± 0.35	2.73 ± 0.49	0.23
2.Geometric distortion	3.97 ± 0.15(whole) 3.95 ± 0.21(lesion)	3.26 ± 0.76(whole) 3.64 ± 0.48(lesion)	< 0.01 < 0.01
3.Ghosting artifacts	3.92 ± 0.26	3.02 ± 0.55	< 0.01
4.Overall imaging quality	4.85 ± 0.35	4.16 ± 0.69	< 0.01
Quantitative parameters			
1.SNR (b = 1000s/mm^2^)			
Lesion	102.3 ± 32.2	493.7 ± 241.6	< 0.01
Brainstem	59.1 ± 15.5	289.8 ± 140.9	< 0.01
2.Contrast	1.79 ± 0.35	1.62 ± 0.44	=0.005
3.CNR (b = 1000s/mm^2^)	4.33 ± 2.8	2.7 ± 2.6	< 0.01

Note: *SNR* Signal to noise ratio, *CNR* Contrast to noise ratio

### Quantitative analysis of image quality

Comparison of the evaluated quantitative image parameters between TGSE and RESOLVE showed significant differences between the two groups. TGSE BLADE DWI produced a significantly lower SNR (*P* < 0.001) and higher parameter values (both contrast and CNR (*P* < 0.001)) than were found for RESOLVE DWI. The results of the statistical analysis are as follows (Table 2): SNR of cholesteatoma, 102.3 ± 32.2 versus 493.7 ± 241.6, respectively (*P* < 0.001); SNR of brainstem, 59.1 ± 15.5 versus 289.8 ± 140.9, respectively (*P* < 0.001); contrast, 1.79 ± 0.35 versus 1.62 ± 0.44, respectively (*P* = 0.005); and CNR, 1.62 ± 0.44 versus 2.7 ± 2.6, respectively (*P* < 0.001).

**Fig. 3:**
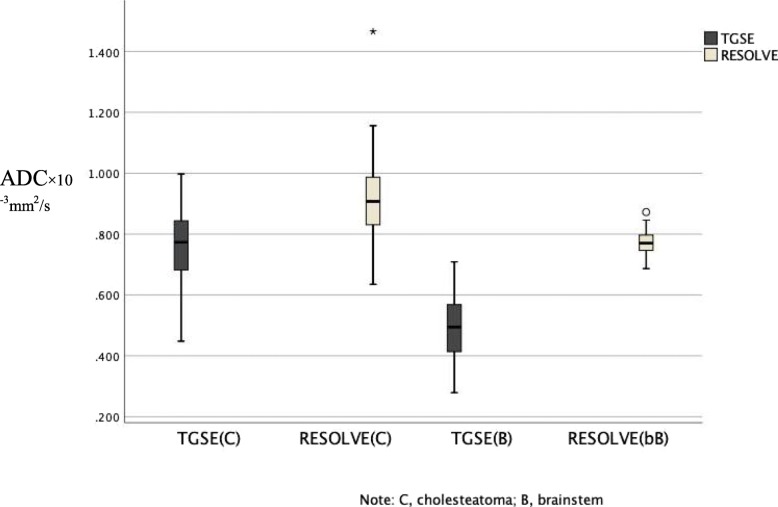
Box plot of the ADC of the cholesteatoma and brainstem showing significant differences in the ADC between TGSE and RESOLVE. The ADC values of the cholesteatoma measured on TGSE and RESOLVE DWI were 0.763 × 10^− 6^ mm^2^/s and 0.928 × 10^− 3^ mm^2^/s, respectively. The ADC values of the brainstem measured on TGSE and RESOLVE DWI were 0.498 × 10^− 3^ mm^2^/s and 0.773 × 10^− 3^ mm^2^/s, respectively

In terms of the measurement and evaluation of the ADC, values were measured in 40 cases, as the lesions were too small to be measured on the ADC maps in 2 cases. Excellent interreader agreement was obtained. Detailed interreader ICCs are shown in Table 3. All ADCs were measured twice by the two observers, and the average values were taken as the basis for further statistical analysis. As shown in Table 3, there was a significant difference in the ADC of cholesteatoma between TGSE BLADE and RESOLVE DWI (*P* < 0.01). The mean ADC of the cholesteatoma measured on TGSE (0.763 × 10^− 3^ mm^2^/s) BLADE DWI was significantly lower than that measured on RESOLVE (0.928 × 10^− 3^ mm^2^/s) DWI (*P* < 0.01). Similarly, the ADC of the brainstem measured on TGSE (0.498 × 10^− 3^ mm^2^/s) BLADE DWI was lower than that measured on RESOLVE (0.773 × 10^− 3^ mm^2^/s) DWI (*P* < 0.01). The box plot in Fig. 3 shows the distributions of the lesion and brainstem ADCs measured on TGSE BLADE and RESOLVE DWI. There was no significant difference in ADC values between the 5 patients with cholesteatoma of the external auditory canal and the 35 patients with cholesteatoma of the middle ear in the TGSE (*P* = 0.236) or RESOLVE (*P* = 0.127) images.

**Table 3:** Comparison of ADC values between two observers

Location	ADC (mean/×10^− 6^ mm^2^/s)			Inter-reader variability (ICC)
	TGSE	RESOLVE	*P* value	TGSE RESOLVE
Cholesteatoma	0.763 ± 0.104	0.928 ± 0.141	< 0.01	0.637 0.911
Brainstem	0.498 ± 0.103	0.773 ± 0.043	< 0.01	0.885 0.759

Note: *ADC* Apparent diffusion coefficient, *ICC* Intra-class correlation coefficient

**Fig. 4:**
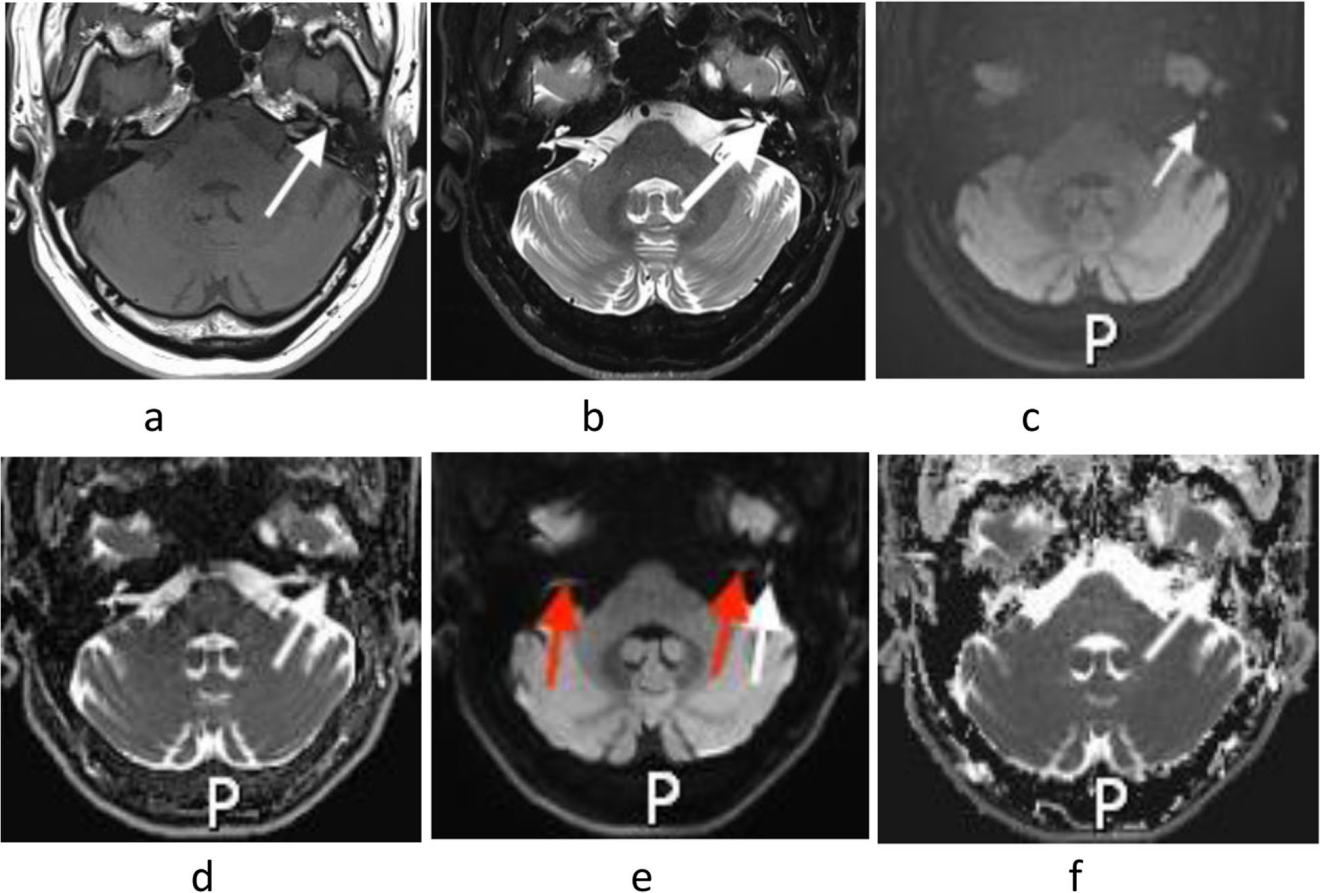
A 44-year-old male with a small cholesteatoma (1.9 mm width) in the left tympanic cavity (white arrow). **a**: Axial T1WI. **b**: Axial T2WI showing the structure of the small cholesteatoma (white arrow). **c**: Axial TGSE BLADE DWI (b1000) clearly showing a markedly high signal intensity for the small cholesteatoma (white arrows) without artifacts. **e**: Axial RESOLVE DWI (b1000) showing the high signal intensity of a small lesion (white arrow) with light geometric distortion and the bilateral middle ear mastoid process with a few artifacts (red arrow). **d**, **f**: The ADC values on TGSE BLADE and RESOLVE DWI were 0.737 × 10^− 3^ mm^2^/s and 0.984 × 10^− 3^ mm^2^/s, respectively

In this study, the measurement results in terms of lesion size were evaluated in 42 patients and showed that TGSE BLADE DWI showed small lesions more clearly than was achieved by RESOLVE DWI. Compared with RESOLVE, TGSE had much better image quality at the air-bone interface (nasal sinus, middle ear, mastoid) and significantly fewer ghosting artifacts and distortion. Furthermore, as shown in Fig. 4, axial TGSE BLADE DWI could completely and clearly show a small lesion (1.9 mm in width) located in the left tympanic cavity with less geometric distortion than was observed using RESOLVE DWI.

## Discussion

DWI is increasingly applied for the evaluation of various diseases in many areas of the body. Conventional DWI (SS-EPI) is often used in head and neck diseases; however, due to interference by the T2* blurring effect and the susceptibility artifacts of various tissues produced by nonmovement, the image quality of conventional DWI (SS-EPI) is usually not satisfactory [9, 28]. Many studies [8, 29, 30] have concluded that the size limit of cholesteatoma on EPI DWI is 5 mm and that smaller cholesteatoma lesions are easily missed on DW EPI. Compared with conventional SS-EPI DWI, RESOLVE has significantly better image quality due to its low susceptibility-based image distortion and T2* blurring and its robust correction for motion-induced phase artifacts [31]. RESOLVE DWI is therefore more widely used in head and neck diseases than SS-EPI DWI is. However, RESOLVE still has some shortcomings that need to be resolved; these include image artifacts and distortion (air-bone interface) that cannot be completely eliminated and a low diagnostic rate of small lesions (< 2.5 mm) [6, 13–15, 32].

To the best of our knowledge, TGSE is a new technique, and the use of DWI in head and neck diseases has not previously been reported in the literature. The basic imaging principles of the TGSE BLADE technique were introduced by Li et al. [33]. In this study, compared to RESOLVE DWI, TGSE BLADE DWI significantly improved the image quality in cases of cholesteatoma by reducing susceptibility artifacts, distortion and blurring when applied at the same scanning time (3 min 46 s). Moreover, TGSE BLADE DWI may be more valuable than RESOLVE DWI for the diagnosis of small cholesteatoma lesions (< 2 mm).

Qualitative analysis of image quality showed that almost no geometric distortion or ghosting artifacts were observed in the TGSE images, while the RESOLVE images contained obvious geometric distortion, mostly in the nasal cavity and mastoid, in 8 of 42 (20%) cases. Moreover, serious artifacts were observed in the RESOLVE images in 5 (12%) cases. Hu [21] et al. also demonstrated that TGSE BLADE DWI produced less geometric distortion in the brain and signal pile-up in highly susceptible areas than was found for conventional SE-EPI. The image quality of TGSE BLADE has also been significantly improved. In the quantitative analysis of image quality, TGSE BLADE DWI produced higher contrast and a higher CNR than was observed for RESOLVE DWI. These were prospective results due to the lack of previous reports on TGSE BLADE DWI.

This study shows that there is a significant difference in the ADC between the TGSE BLADE and RESOLVE sequences, with a significantly lower ADC of the cholesteatoma (*P* < 0.01) and brainstem (*P* < 0.01) found when using TGSE BLADE DWI than RESOLVE DWI. The average ADC for cholesteatoma on RESOLVE DWI was 0.928 × 10^− 3^ mm^2^/s, which is consistent with the cholesteatoma ADC (0.7–1.0 × 10^− 3^ mm^2^/s) previously reported in the literature [6, 34, 35]. The mean ADC of the cholesteatoma and brainstem on TGSE BLADE DWI was 0.763 × 10^− 3^ mm^2^/s and 0.498 × 10^− 3^ mm^2^/s, respectively. These findings demonstrate that the ADC obtained in our study should provide an auxiliary basis for more clinical applications of TGSE BLADE DWI in the future.

However, there are also some limitations to our study: the number of patients included in this study was relatively small, and the error caused by manual measurement could not be eliminated. This may have affected the accuracy of the true range of the ADC on TGSE BLADE DWI. Moreover, TGSE BLADE DWI is not without its disadvantages. The overall image SNR of TGSE is slightly lower than that achieved by RESOLVE, mainly because placement of the gradient echo with T2* decay effects in the center of k-space reduces the image quality of TGSE, consistent with a previous pediatric brain study reported by Ui [28] et al.

## Conclusion

In conclusion, TGSE BLADE DWI produced better image quality than was achieved by RESOLVE DWI in the diagnosis of cholesteatoma. TGSE BLADE DWI was also superior to RESOLVE DWI in terms of image distortion, artifacts and lesion conspicuity. In addition, TGSE BLADE DWI appears to be more effective than RESOLVE DWI in detecting small lesions.

## Data Availability

The datasets used and analyzed in the current study are available from the corresponding author on reasonable request.

## Abbreviations

ADC: Apparent diffusion coefficient
SNR: Signal-to-noise ratio
CNR: Contrast-to-noise ratio
SAR: Specific absorption rate
ROI: Region of interest
ICC: Intraclass correlation coefficient
